# Mental health literacy among primary healthcare workers and its implications on detecting common mental health disorders across five geopolitical zones in Nigeria

**DOI:** 10.4314/gmj.v59i4.7

**Published:** 2025-12

**Authors:** Joshua Falade, Usen J Silas, Olusola Falade

**Affiliations:** 1 Department of Mental Health, University of Medical Sciences, Ondo, Ondo State, Nigeria; 2 Department of Community Health, Wesley University, Ondo, Ondo State, Nigeria; 3 School of Nursing, Asubiaro, Osogbo, Osun State, Nigeria

**Keywords:** Mental Health Literacy, PHC workers, Nigeria, Mixed Study

## Abstract

**Objective:**

To determine mental health literacy levels and their predictors among primary health care workers across Nigeria's five geopolitical zones, and to identify common patient presentations and rates of suspected psychological illness.

**Design:**

A cross-sectional, mixed-methods study using a census method

**Setting:**

The National Refresher Course Centre of the National Association of Community Health Practitioners of Nigeria, held at Wesley University, Ondo State.

**Participants:**

66 members of the National Association of Community Health Practitioners of Nigeria.

**Interventions:**

A semi-structured questionnaire to collect socio-demographic data and Focus Group Discussions were also conducted among Community Health Practitioners in each zone.

**Main outcome measures:**

Mental health literacy and the predictors

**Results:**

Mental health literacy among primary health care workers was low, with only 12.1% demonstrating above-average knowledge, mainly associated with longer work experience and a family history of mental illness. Patients commonly presented with physical complaints and symptoms such as fever, headache, insomnia, weight loss, and weakness, while underlying mental health conditions like anxiety, depression, and substance use disorders were often unrecognised. Routine mental health assessment was rare, and referrals to psychiatric services were infrequent, with most workers reporting only two to three referrals in the past year.

**Conclusion:**

The mental health literacy of primary health care workers in Nigeria is critically low, highlighting an urgent need for extensive capacity building to improve the detection of psychological illness for comprehensive healthcare delivery.

**Funding:**

None declared

## Introduction

Mental health remains a crucial component of overall health and well-being, but has been largely neglected in Africa.^1^ The World Health Organisation (WHO) estimates that approximately 450 million people globall suffer from mental disorders, with one in four individuals likely to experience a mental illness during their life-time.^2^ In Nigeria, a significant gap exists between mental healthcare needs and available services. Although there have been improvements in health policies and universal health coverage efforts, mental health service delivery continues to face persistent challenges.^3^ These challenges include inadequate policy frameworks, insufficient legislation, limited funding, poor research capacity, lack of training, and weak integration of mental healthcare into primary healthcare.^4^ Moreover, poverty, low educational levels, and poor mental health literacy among primary healthcare workers in Sub-Saharan Africa exacerbate the mental health treatment gap.^4^ Limited healthcare investment and the lingering effects of COVID-19 further threaten to widen this gap.

Nigeria's healthcare system operates through three tiers: primary, secondary, and tertiary care. Primary healthcare (PHC) facilities, largely managed by local government authorities in collaboration with state governments and international donors, serve as the entry point to healthcare for most Nigerians, particularly in rural areas.^6^ Cases requiring specialised care are referred to secondary and tertiary centres. Globally, integrating mental healthcare into PHC has been a longstanding strategy.^7^

Countries such as Argentina, Australia, India, Saudi Arabia, South Africa, Uganda, and the United Kingdom have successfully embedded mental health into primary care, recognising it as essential to comprehensive healthcare delivery.^8^ Integrating mental health into PHC increases accessibility, affordability, and cost-effectiveness while upholding human rights and improving health outcomes.^9^ This underscores the importance of expanding mental healthcare into rural, community-based rehabilitation centres supported by well-coordinated primary, secondary, and tertiary networks.

The concept of Mental Health Literacy (MHL) originally focused on knowledge and beliefs about mental disorders that facilitate their recognition, prevention, and management. Initially, MHL targeted adult populations, particularly healthcare professionals, to raise mental health awareness.^10^ Over time, MHL evolved into a broader framework encompassing education and interventions to promote positive mental health within entire communities.^11^ While health literacy is associated with economic prosperity (WHO, 2013), poor MHL among PHC workers contributes to the mental health treatment gap and disease burden in Sub-Saharan Africa.^12^

Improving MHL among PHC workers is critical for integrating mental health services and achieving Sustainable Development Goals for global mental health.^13^ However, MHL transcends mere knowledge acquisition; it must be understood within specific cultural contexts. Cultural attitudes and traditional beliefs significantly shape perceptions of mental illness and influence help-seeking behaviours.^14^ The Executive Director of the National Primary Healthcare Development Agency (NPHCDA) emphasised that 70% of Nigerians reside in rural areas where PHC centres are often the only available health facilities. Alarmingly, over 70% of PHCs lack essential infrastructure, medications, and basic utilities.^15^

The burden of mental disorders in Nigeria is substantial. A study in southwestern Nigeria reported prevalence rates of 5.5% for depression, 3.5% for generalised anxiety, and 1.2% for combined depression/anxiety symptoms, with 20.9% comorbidity between depression and anxiety.^16^ Among retirees in North Central Nigeria, loneliness, depression, anxiety, and anxious depression prevalence rates were 21.8%, 52.0%, 27.7%, and 20.5%, respectively.^17^ In southeastern Nigeria, 70% of respondents experienced depressive disorders, 52.3% had substance use disorders (particularly tranquillisers at 34.9% and stimulants at 15.8%), and 85.3% suffered from anxiety disorders.^18^

To address this burden, PHC workers require professional training in recognising and managing mental health problems such as depression and anxiety. Culturally informed mental health literacy interventions are essential for effective mental healthcare delivery because cultural beliefs strongly influence community attitudes toward mental illness. Despite the crucial role PHC workers could play in improving mental health literacy within communities, research on MHL and its determinants among PHC professionals in Nigeria remains limited, indicating a clear need for further studies.

The aim of the study was to determine mental health literacy and its predictors among primary health care workers, and to identify common patient presentations and the rate of suspected psychological illness across the five geopolitical zones of Nigeria.

## Methods

### Study Location

The study was conducted at the National Refresher Course Centre of the National Association of Community Health Practitioners of Nigeria, held at Wesley University, Ondo, Ondo State, Nigeria. The program brought together Community Health Extension Workers (CHEWs) practising at Primary Health Care Centres (PHCs) in Nigeria.

### Study Population

The study populations were Junior CHEWs and Senior CHEWs practising in the Nigerian Primary Health Care Centres. The study was conducted between the 12th and 17^th^ of August 2024.

### Study Design

The study employed a cross-sectional design with mixed qualitative and quantitative methods. Census method was used with the use of a questionnaire to collect the socio-demographic. A focus group discussion was conducted among health workers from each geopolitical zone. CHEWs who practised in the past year were included in the study.

### Instruments

#### Mental Health Literacy Scale

The Mental Health Literacy Scale (MHLS) is a 35-item questionnaire looking at the respondents' understanding of mental health. The MHLS is a one-dimensional measurement scale with 35 items and six attributes based on Jorm's six MHL attributes.^19^ The scale items were generated using a combination of adaptation of existing MHL items, descriptors from the Diagnostic and Statistical Manual of Mental Disorders, DSM-IV-TR, national and international data, and the clinical experience of the authors and their clinical panel, who advised the item generation.^20^ The scale score ranges from 35 to 160, with a higher score implying a higher level of MHL. The scale has the following sections: Recognition of Disorders (8 items measured on a 4-point Likert scale), Knowledge of Risk Factors and Causes (2 items measured on a 4-point Likert scale), Self-Treatment Knowledge (2 items measured on a 4-point Likert scale), Knowledge of Professional Help Available (3 items measured on a 4-point Likert scale), Knowledge of How to Seek Mental Health Information (4 items measured on a 5-point Likert-scale) and Attitudes that Promote Recognition and Appropriate Help-Seeking (16 items measured on a 5-point Likert scale), with items 10, 12, 15 and 20–28 as reverse-scored items. 11.^21^ The rigour with which the MHLS was developed and its subsequent psychometric properties have made it the most reliable and validated instrument for assessing MHL The scale showed internal consistency with a Cronbach's alpha of 0.79 in a Nigerian study.^21^

In addition, the MHLS is the only available instrument to measure all aspects of MH. In this study, the scores across all aspects were summed. Respondents who scored below 50% of the total mark were classified as below-average, while respondents who scored 50% and above of the total mark were classified as above-average. A pilot study conducted among 50 Community Health Extension workers in the Ondo West Local Government Area who were not part of the study revealed that the average time to complete the questionnaire was 4 minutes. Test-retest reliability after two weeks was 0.82, Internal consistency was α = 0.81, and two independent psychiatrists validated the instrument.

#### Procedure

Having retrieved the lists of the Primary Health Workers who attended the annual refresher course from the Chairman of the Local Organising Committee, the objective of the study was discussed with the respondents. Confidentiality was assured, and the study's benefits were explained. Consent for participation was obtained from respondents who met the inclusion criteria.

The respondents completed self-administered questionnaires, which were collected by the researchers and research assistants. Focus Group Discussions (FGDs) were conducted with primary health care workers from each geopolitical zone of Nigeria, in separate classes at different times.

### Data Analysis

The Statistical Package for Social Sciences (SPSS version 21) was used for Data analysis. The socio-demographic details of respondents were reported using descriptive statistics, including frequencies and percentages. Chi-square and t-tests were employed to identify factors significantly associated with Mental Health literacy among respondents. The confidence interval was set at 95%. Statistical significance was considered at a p-value less than 0.05. The coding process was used to identify the themes. NVivo 14 was used to code the transcript.

### Ethical approval

Ethical approval was obtained from the Ethics and Research Ethics Committee of the University of Medical Sciences Teaching Hospital, Ondo City, Ondo State (UNIMEDTH/REC/24/025).

## Results

A total of 66 respondents participated in the study; 60.6% were female, the majority (90.9%) were married, and 42.2% were between grades 8 and 12. Most of them were satisfied with the job, while 78.8% and 80.0% did not have personal and family history of mental illness, respectively. The average age of the participants was 41.1 years (±6.3). On average, they saw 20.01(±13.0) patients per day and had 8.23 years (±3.80) of practice experience. Their average monthly income was 71,420 Naira (±41,410), and they worked an average of 9.79 hours per day (±5.40). (Table 1)

**Table 1:** Characteristics of study participants

Variable	Category/Value	Frequency (n)	Percentage (%)
Gender	Female	40	60.6
	Male	26	39.4
Marital status	Single	6	9.1
	Married	60	90.9
GL CAT	0–7	26	39.4
	8–12	28	42.4
	13–17	12	18.2
Work satisfaction	No	22	33.3
	Yes	44	66.7
Place of practice zone			
	South West	14	21.2
	South East	4	6.1
	South South	26	39.4
	North West	12	18.2
	North Central	10	15.2
Family history of MI			
	Negative	52	78.8
	Positive	14	21.2
Personal history of MI	Negative	53	80.3
	Positive	13	19.7
Average Age	Mean ± SD	41.1 ± 6.3	
Average patients seen per day	Mean ± SD	20.01 ± 13.0	
Average years of practice	Mean ± SD	8.23 ± 3.80	
Average monthly income (Naira)	Mean ± SD	71.42 ± 41.41	
Average number of hours per day	Mean ± SD	9.79 ± 5.40	

### Sociodemographic characteristics of the respondents

Among the respondents, 12.1% had above-average mental health knowledge, while family history of Mental illness, higher grade level and increased years of experience were associated with above-average mental health literacy (Tables 2 and 3).

**Table 2:** The association between sociodemographic characteristics and Mental Health Literacy Level using Chi-square

Variables	Below average	Above average	*X* ^2^	Df	*P-Value*
**Gender**					
**Female**	38(95.0%)	2(5.0%)	4.834	1	0.050
**Male**	20(76.9%)	6(23.1%)			
**Marital status**					
**Single**	4(66.7%)	2(33.3%)	2.788	1	0.095
**Married**	54(90.0%)	6(10.0%)			
**GL CAT**					
**0 TO 7**	24(92.3%)	2(7.7%)	6.199	2	**0.045**
**8 T0 12**	26(92.9%)	2(7.1%)			
**13 TO 17**	8(66.7%)	4(33.3%)			
**Are you satisfied with the work you do**					
**No**	18(81.8%)	4(18.2%)	1.138	1	0.266
**Yes**	40(90.9%)	4(9.1%)			
**Family history of MI**					
**Negative**	50(96.2%)	2(3.8%)	15.759	1	**0.001**
**Positive**	8(57.1%)	6(42.9%)			
**Personal history of MI**					
**Negative**	47(88.7%)	6(11.3%)	0.162	1	0.687
**Positive**	11(84.6%)	2(15.4%)			

**Table 3:** Association between the sociodemographic characteristics and mental health literacy using the t-test

Variables	Below Average	Above Average	T Test	Df	*P Value*
**Age**	40.66±6.50	44.00 ±2.92	4.16	64	0.158
**average patients per day**	20.03±13.71	12.75±2.96	9.01	64	0.142
**year of practice**	7.002±1.52	17.00±4.00	10.74	64	***≤0.001***
**Average monthly income in Naira**	70,024±38,061	80,000±61,875	4.92	64	0.534
**number of work hours**	10.10±5.73	7.50±0.53	5.61	64	0207

### Qualitative Analysis

The resulting themes are described in the summary of the research findings. The coding process identified seven primary themes.

### Common patient presentations at the health Centre

Based on the interview responses, several common health conditions are frequently present at the health centre. These conditions primarily involve symptoms such as fever, headache, crawling sensations, diarrhoea, vomiting, body pain, and generalised body weakness. Fever and different body pains were the most frequent symptoms present at the health Centre, some of the presented symptoms are also headache, insomnia, weight loss and poor feeding and frequent urination.

*“The common presentations are Fever, headache, body pain, crawling sensations”* Respondent 7, South South

*“Signs and Symptom I see is heat sensations in the head, pains, fever is also there, we have shivering also in terms of malaria abdominal tenderness, weight loss, poor feeding”* Respondent 2, Southeast

*“Vomiting, hotness in their body, weakness, body pains.”*

*“Some symptoms are dizziness, fever, adults complain waist pain, knees pain”* Respondent 12, South south

*“Some may come with frequent urination, excessive thirst for hunger, pains, tiredness”* Respondent 4, Southeast

### Common diagnoses made by primary health care workers

The most common diagnosis was malaria; the majority of the respondents were diagnosed as having malaria after the presentation. Some of the respondents mentioned typhoid fever and, in very rare cases, hypertension, pneumonia, anaemia, tuberculosis and sometimes HIV cases. One of the respondents said, *“We use to have Postpartum psychosis”*

*“we are going to conduct malaria test for such person”* Respondent 1, North central

*“Malaria, typhoid fever”* Respondent 5, Northwest

*“Hypertensive, malaria”* Respondent 6, Southeast

*“Malaria, typhoid, sometimes HIV cases, tuberculosis once in a while”* Respondent 2, North Central

*“pneumonia, malaria”* Respondent 4, Southwest

### Awareness about anxiety, depression and substance use disorder

Based on the awareness of psychological disorders, a significant proportion of the primary health workers are not aware of anxiety, depression and substance use disorder among patients; likewise, a few proportions of them are aware of these disorders.

*“No idea”* Respondent 4, Southwest

*“No”* Respondent 3, Southsouth

*“Yes, this not the first time actually”* Respondent 1, Northwest

### Identification and Suspicion of Mental Health Disorders

Regarding how these disorders can be identified in patients, numerous symptoms that are traceable to it were mentioned by the primary health workers. Some of the respondents said, mental health patients become aggressive and violent, there are changes in some of their behaviors as well, it was stated by one of them that people who abuse substance abuse disorders are most likely going to behave irrational Another primary health worker said depression could be identified when their blood pressure is being checked. It is high, such patients could be engaged in discussion as to open up on what they are going through. A very notable trait for people with depression was isolation; it was stated that those patients just gradually dissociate themselves from what they normally take pleasure in doing, and in so doing, they start to have the thought of suicide. One of the health workers interviewed also noted that it can be detected from the patient's fear and voice. It was also stated by one of the interviewees that if they couldn't perform some changes at the physiological stages or walk around from place to place, also in their manner of talking and socializing with people can be traced to substance abuse, Still talking about substance abuse, one of the respondents said it can be noticed during operation, when such people are giving sedatives which ought to make them relax, the person's body struggles and refuses the drugs.

*“Anxiety disorder is whenever one is so anxious with what he or she wants to do, they won't concentrate”* Respondent 4, North Central

*“Substance abuse: any person that is taking certain drugs or drinking or smoking if he is behaving irrationally we trace that person to what he is taking”* Respondent 1, North Central

*Depressive if the person is maybe you do Blood Pressure for the person and advise him what really happened to the person through that statement”* Respondent 3, South south

*“Actually for depression, you find the person isolating himself start thinking of how can he even kill himself in terms of depression. While in anxiety, will be having fear, is there anything that can affect him? While in terms of substance abuse some people will depend on drug or hard substance to some extent they will not live without taking it.”* Respondent 5, Northwest

*“Let me start with the substance abuse, sometimes to suspect will come from if some of the activities they are not able to perform changes in the physiological stages in individuals or they should walk from one place to another, so the reaction can also indicate which is one of substance abuse and also the manner of touching and socialization to environment. But depression comes with Isolation, living quietly in an area the person will not actively involved in activities probably there is something disturbing the person. Anxiety mostly comes with scare, you see people eager, urge expecting something to come.”* Respondent 2, Northwest

*“simply means when somebody is afraid of many things in his or her environment, if you see a patient before he or she talks one or two anything you will notice from the voice”* Respondent 2, North Central

*“In talking about substance abuse, when somebody is using drugs maybe during the operation the drug will not be able to enter that person body, if you're giving that person drugs, all this sedative that supposed to relax that person muscle the person will still be struggling, you will now say that the person is into drugs like alcohol”* Respondent 3, North central

### Frequency of psychological Symptom Inquiry from patients

Most of the respondents do not always inquire about anxiety, depression and substance abuse symptoms from their patients, while some of them do occasionally. One of the primary health workers said they usually inquire during health education and maternity care, some of the health workers said they don't ask at all while very few of them ask regularly.

*“Not always”* Respondent 2, Northwest

*“Not frequently”* Respondent 5, Southeast

*“Not often”* Respondent 3, South south

*“No unless they present it”* Respondent 5, Southwest

*“During health education and during maternity care. I do that occasionally.”*

*“Regularly”* Respondent 5, South south

*“Each time we have in patient and outpatient”* Respondent 4, Northcentral

### Number of referred patients to Psychiatrists in the Past Year

The number of patients who have been referred to psychiatrists by the primary health workers varies. The majority of the respondents have not referred any patients, while some have referred just one in the past year. The highest number of patients referred to the psychiatrists in the past year was two and three.

*“None”* Respondent 3, Southeast

*“One, when I was in school during my second practice ”* Respondent 2, North Central

*“I have referred about two or three”* Respondent 1, Southwest

## Discussion

This discussion section analyses the critically low mental health literacy (MHL) found among Nigerian Primary Healthcare (PHC) workers, contextualising it within the challenges of somatic symptom presentation, the national mental health burden, and overarching systemic healthcare failures. The analysis is structured into three core themes: the diagnostic dilemma of somatic presentations, the vast treatment gap exacerbated by systemic issues, and the factors associated with variations in MHL within the workforce.

The study identifies a fundamental diagnostic paradox. PHC workers reported common patient symptoms such as generalised body pain, fatigue, headaches, a crawling sensation, and insomnia, with malaria being the most frequent diagnosis. Crucially, most workers did not associate these physical symptoms with psychological illness. However, conditions like depression, somatoform disorders, and anxiety disorders are known to present primarily through such somatic symptoms. This creates a significant challenge in malaria-endemic Nigeria, where the clinical training of PHC workers is heavily oriented toward diagnosing and treating endemic diseases like malaria and typhoid, which share a symptomatic overlap with mental health disorders.^22^

This phenomenon is not unique to Nigeria; somatic symptoms account for over half of all primary care visits globally and are often chronic, recurrent, and medically unexplained.^23^ Internationally, a majority of patients with depression or anxiety present to primary care with physical complaints rather than articulating psychological distress.’ In fact, two-thirds of patients with major depression present exclusively with somatic complaints.^24^, This pattern of presentation is common across cultural settings.^25^ Symptoms of anxiety disorders (chest pain, palpitations, dizziness) and substance abuse (nausea, tremors from withdrawal) further complicate diagnosis, as they closely mimic purely medical conditions.^26^, The discussion emphasises that the presence of multiple, persistent, or medically unexplained somatic symptoms is a powerful indicator of an underlying psychological disorder. There is a strong “dose-response” relationship, whereby a higher number of physical complaints significantly increases the likelihood of a comorbid depressive or anxiety disorder.^27^,

The failure to detect mental illness at the primary care level directly widens Nigeria's severe mental health treatment gap. The study's finding of extremely low detection rates stands in stark contrast to the documented national burden, where an estimated 20% of the population (approximately 40 million people) is affected by mental illness.^28^ This includes about 7 million people with depressive disorders and 4.9 million with anxiety disorders.^29^,

This chasm between prevalence and detection is driven by a confluence of factors. Primarily, PHC workers lack the necessary training and awareness to recognise the psychological origins of physical symptoms.^30^ Furthermore, profound societal stigma and cultural beliefs that attribute mental illness to supernatural causes lead to both patient concealment and provider oversight.^31^ These challenges are massively exacerbated by a collapsing healthcare system. The “Japa Syndrome”, the mass exodus of skilled health workers, has devastated the PHC sector, which was already crippled by poor infrastructure, derogatory working conditions, and a critical shortage of staff, especially in rural areas where 52% of the population lives.^32^ This systemic crisis results in rushed consultations, a complete lack of diagnostic tools, poor integration of mental health services, and non-existent referral pathways, creating an environment where the detection of mental illness is nearly impossible.^33^

The mental health literacy among the respondents was found to be abysmally low, which directly explains the low suspicion of psychological illness and the minimal number of referrals to psychiatrists^34^, This contrasts with findings from PHCs in other African nations, such as South Africa and Kenya, suggesting a particularly acute problem in Nigeria.^35^

Despite the overall poor MHL, the study identified two key predictors of marginally better knowledge. First, a family history of mental illness was significantly associated with above-average MHL. This personal exposure acts as an informal training mechanism, fostering greater awareness, reducing personal stigma, encouraging open discussion, and motivating individuals to proactively seek information. Second, higher professional grade level and more years of experience were also positive predictors. Long-term practitioners likely acquire skills through experiential learning from past encounters and mentorship from senior colleagues. This accumulated practical knowledge provides a broader understanding of complex symptom patterns and improves communication skills, making patients feel more comfortable disclosing psychological distress. However, the discussion posits that these factors, reliant on personal circumstance and slow professional accumulation, are inadequate solutions for a national crisis requiring structured, systemic intervention.

Urgent action is required from Nigerian health policymakers and training institutions to integrate comprehensive, practical mental health training into the mandatory curriculum for all Primary Healthcare workers. This training must focus on recognising the somatic symptoms of common psychological disorders and developing clear, functional referral pathways to specialists.

## Conclusion

This study concludes that the mental health literacy of primary healthcare workers in Nigeria is critically low, severely limiting the detection of psychological illness at the primary care level. This deficiency represents a major barrier to achieving comprehensive healthcare and necessitates immediate capacity-building interventions.
